# N-Formyl Methionine Peptide-Mediated Neutrophil Activation in Systemic Sclerosis

**DOI:** 10.3389/fimmu.2021.785275

**Published:** 2022-01-05

**Authors:** Runa Kuley, Ryan D. Stultz, Bhargavi Duvvuri, Ting Wang, Marvin J. Fritzler, Roger Hesselstrand, J Lee Nelson, Christian Lood

**Affiliations:** ^1^ Department of Medicine, Division of Rheumatology, University of Washington, Seattle, WA, United States; ^2^ Department of Biochemistry and Molecular Biology, University of Calgary, Calgary, AB, Canada; ^3^ Department of Clinical Sciences Lund University, Section of Rheumatology, Lund, Sweden; ^4^ Clinical Research Division, Fred Hutchinson Cancer Research Center, Seattle, WA, United States

**Keywords:** systemic sclerosis, autoimmunity, neutrophils (PMNs), neutrophil extracellular traps (NET), mitochondrial peptides, clinical biomarkers

## Abstract

Exaggerated neutrophil activation and formation of neutrophil extracellular traps (NETs) are reported in systemic sclerosis (SSc) but its involvement in SSc pathogenesis is not clear. In the present study we assessed markers of neutrophil activation and NET formation in SSc patients in relation to markers of inflammation and disease phenotype. Factors promoting neutrophil activation in SSc remain largely unknown. Among the neutrophil activating factors, mitochondrial-derived N-formyl methionine (fMet) has been reported in several autoinflammatory conditions. The aim of the current study is to assess whether SSc patients have elevated levels of fMet and the role of fMet in neutrophil-mediated inflammation on SSc pathogenesis. Markers of neutrophil activation (calprotectin, NETs) and levels of fMet were analyzed in plasma from two SSc cohorts (n=80 and n=20, respectively) using ELISA. Neutrophil activation assays were performed in presence or absence of formyl peptide receptor 1 (FPR1) inhibitor cyclosporin H. Elevated levels of calprotectin and NETs were observed in SSc patients as compared to healthy controls (p<0.0001) associating with SSc clinical disease characteristics. Further, SSc patients had elevated levels of circulating fMet as compared to healthy controls (p<0.0001). Consistent with a role for fMet-mediated neutrophil activation, fMet levels correlated with levels of calprotectin and NETs (r=0.34, p=0.002; r=0.29, p<0.01 respectively). Additionally, plasma samples from SSc patients with high levels of fMet induced *de novo* neutrophil activation through FPR1-dependent mechanisms. Our data for the first time implicates an important role for the mitochondrial component fMet in promoting neutrophil-mediated inflammation in SSc.

## Introduction

Systemic sclerosis (SSc) is a rare chronic autoimmune disease characterized by vasculopathy, inflammation, and fibrosis of the skin and internal organs. Depending on the distribution of skin fibrosis, SSc is clinically subdivided into limited cutaneous SSc (lcSSc) and diffuse cutaneous SSc (dcSSc) (1, 2). The pathogenesis of SSc is complex and the exact etiology of the disease is still unknown. The role of the adaptive immune system with autoreactive T and B cells producing autoantibodies in SSc pathogenesis has been well established (3, 4). Additionally, several studies have suggested the involvement of the innate immune system in early pathogenic events in SSc but this has not been explored in detail (5).

Among the innate immune cells, neutrophils are prominent contributors of inflammation in several autoimmune diseases including rheumatoid arthritis (RA) and systemic lupus erythematosus (SLE), and have also been implicated in SSc pathogenesis (6–9). While neutrophils play a protective role against invading pathogens through reactive oxygen species (ROS) production, phagocytosis and formation of neutrophil extracellular traps (NETs) (10, 11), unrestrained neutrophil activation may lead to inflammation, immune dysregulation and tissue damage. Neutrophils are the most abundant immune cells in the circulation, and in SSc patient’s blood neutrophil counts are elevated and correlated with disease severity (12). Neutrophils were also increased in skin biopsies obtained from forearm lesions in SSc patients compared with controls (13). Moreover, neutrophil involvement in induction of endothelial cell apoptosis, an early event leading to fibrosis, has been reported in SSc (14). These studies indicate a critical pathogenic or modulatory role of neutrophils in SSc.

Markers of neutrophil activation, such as elevated levels of circulating calprotectin and NET formation have been previously reported in several autoimmune disorders and evolved as excellent biomarkers for inflammatory processes (6, 8, 15). Calprotectin (S100A8/A9) is a calcium-binding protein abundantly expressed in neutrophils and is essential for initiating immune responses to non-infectious inflammatory processes (16, 17). NET formation is the key feature of neutrophil cell death in which nuclear DNA is extruded together with cytoplasmic and granular contents in web-like structures that can function to entrap and eliminate extracellular pathogens (18). Elevated levels of neutrophil activation markers during an inflammatory response may be detrimental and cause bystander injury that could be perpetual or non-resolving. Among these markers, separate studies have found increased soluble levels of calprotectin, an acute phase protein expressed by neutrophils and NETs in SSc patients and associations with SSc disease manifestations were also reported in these studies (19–22). Additionally, filaments of DNA coming from elastase positive cells, such as neutrophils can be seen in SSc skin (23). Although these studies point to a role of neutrophils in SSc, a comprehensive study of neutrophil activation markers in the circulation of SSc patients and its contribution to inflammation and disease phenotype needs to be carefully addressed.

So far, factors contributing to neutrophil-mediated activation in a sterile pro-inflammatory environment like SSc remains largely unknown. During inflammation, neutrophils are activated by various molecules, including cytokines, immune complexes (ICs) and damage-associated molecular patterns (DAMPs) generated by mitochondrial components (11, 24, 25). We and others have reported mitochondrial extrusion and presence of extracellular mitochondrial components in several autoimmune disorders such as SLE and RA (26–28). Given their prokaryotic origin, mitochondria contain several pro-inflammatory components that can engage neutrophils. Among the mitochondrial protein-derived molecules, N-formyl-methionine (fMet) is a potent neutrophil chemoattractant that can trigger a variety of neutrophil functions leading to tissue damage. fMet acts through formyl peptide receptor-1 (FPR1), which is abundantly expressed on neutrophils and when bound by its cognate ligand induces inflammation (29–32). However, its role remains to be explored in SSc.

In the current study, we first assessed the levels of neutrophil activation markers in two independent SSc cohorts and investigated their association with markers of inflammation and SSc disease phenotype. Secondly, we analyzed levels of fMet in relation to neutrophil activation levels. Finally, we assessed whether fMet could contribute to neutrophil activation through FPR1-mediated signaling in SSc patients. Briefly, neutrophil activation markers were elevated in SSc patients and associated with a disease phenotype. We also made the novel observation that elevated fMet levels were associated with neutrophil activation in SSc patients. Finally, plasma samples from SSc patients induced *de novo* neutrophil activation through an FPR1-dependent mechanism suggesting FPR1 as a potential novel therapeutic target in these patients to reduce neutrophil-mediated inflammation.

## Materials and Methods

### Patient Cohorts and Ethical Statement

Plasma samples from two independent SSc cohorts were analyzed in the current study. The distributions of the sex, age, ethnicity, disease subgroups, prevalence of anti-centromere, anti-topoisomerase, anti-RNA Polymerase III, anti-survival of motor neuron 1 (SMN1) and anti- U11/12 ribonucleoprotein (RNP-C3) autoantibodies and clinical characteristics among the SSc patients are detailed in **Table 1**.

**Table 1:** Demographic and clinical information on disease and control groups.

Cohort	SSc 1	SSc 2	HC 1	HC 2
Patients (#)	81	20	40	24
Specimen	Plasma	Plasma	Plasma	Plasma
Age in years (Median, range)	47 (20-80)	67 (19-82)	57 (26-71)	35 (18-67)
Disease duration (Median, range)	4.5 (0-29)	8.5 (0-19)	N/A	N/A
Gender (% female)	100	80	85	88
Ethnicity (White, %)	72	95	100	96
lcSSc (%)	31	60	N/A	N/A
dcSSc (%)	65	40	N/A	N/A
ACA+ (%)	23	25	N/A	N/A
Scl70 (%)	20	25	N/A	N/A
RNAPIII (%)	28	15	N/A	N/A
SMN1 (%)	3	N/D	N/A	N/A
p53 (%)	4	N/D	N/A	N/A
Ro52 (%)	16	N/D	N/A	N/A
PM-Scl-75	9	N/D	N/A	N/A
PM-Scl-100	8	N/D	N/A	N/A
Th/To	13	N/D	N/A	N/A
U11/12 RNPC3	16	N/D	N/A	N/A
Skin score	ND	4 (0-43)	N/A	N/A
Telangiectasia (%)	ND	40	N/A	N/A
Digital pitting scar (%)	ND	45	N/A	N/A
NT-pro-BNP	ND	226 (0-1342)	N/A	N/A

lcSSc, Limited cutaneous scleroderma; dcSSc, Diffuse cutaneous scleroderma. No data on lcSSc or dcSSc manifestation in 4% SSc patients from (Cohort I). ACA: Anti-centromere Abs. Scl70: Anti-topoisomerase Abs.

RNAPIII: Anti-RNA Polymerase III. SMN1, Anti-survival of motor neuron 1; U11/12 (RNPC3), Anti-Ribonucleoprotein.

Cohort I comprised 80 SSc patients including 13 who presented for evaluation for autologous hematopoietic cell transplantation (HCT) due to severe SSc and 67 patients were recruited from Rheumatology practices primarily in the Seattle, Washington area, with some patients from Alaska, Montana, Oregon and other states. Patients presenting for HCT had diffuse severe SSc (n=11 autologous HCT, n=2 allogeneic HCT), criteria including modified Rodnan skin score ≥16, SSc 3 years or less from onset of first non-Raynaud symptom, and either FVC or DLCO <70% of predicted, or myocardial disease, or history of proteinuria >500mg/124hr as evaluated by transplant protocols with further details provided for autologous HCT in prior publication (33). Patients recruited from clinical Rheumatology practices were assessed through a combination of medical record review, patient administered questionnaires and, when information was insufficient, direct contact with the rheumatologist consultant. Cohort I also included 40 healthy controls recruited from Seattle WA and the surrounding area.

Cohort II consisted of 20 SSc patients and 24 healthy controls recruited from Skane University Hospital, Lund, Sweden. Age- and gender-matched healthy individuals were recruited to participate in the research studies. Cohort II was included to validate key findings from Cohort I, as well as assess clinical associations. Patients were classified according to the 2013 ACR classification criteria (34) and were stratified as having lcSSc or dcSSc according to the extent of skin involvement. All patients from Cohort II underwent evaluation by ECG, echocardiography, cineradiography of esophagus, high resolution computed tomography of the lungs and pulmonary function tests (spirometry). The patients also had a measurement of serum NT-pro-BNP. During the clinical assessment, the modified Rodnan skin score was used, and clinicians noted the presence or absence of digital pitting scars, ulcers, and telangiectasia. All clinical characteristics are summarized in **Table 1**.

The Cohort I and II used in our study are quite different clinically. The SSc patient population from Cohort I who were being evaluated for HCT is atypical from what is routinely seen in clinic as it is enriched for patients with severe and rapidly progressive SSc. Thus, Cohort I, unlike Cohort II, is skewed to diffuse SSc with more severe disease. In addition, Cohort I differs from Cohort II for ethnicity % and gender % (100% of Cohort I are female) (**Table 1**).

The study was approved by the Institutional Review Board of Fred Hutchinson Cancer Research Center (part of the University of Washington Consortium) and Lund University #2010/544. Informed written consent was obtained from all study participants according to the Declaration of Helsinki.

### ELISA-Based Methods

Levels of circulating calprotectin were analyzed using a commercial ELISA kit according to manufacturer’s instructions (R&D Systems, Minneapolis MN, USA). Circulating NETs were quantified using a myeloperoxidase (MPO)-DNA ELISA, as described previously (6, 26). Briefly, 96-well microtiter plate (Corning) was coated with anti-MPO antibody (4 μg/ml; Bio-Rad Laboratories, Hercules, CA, USA) overnight at 4°C, followed by blocking with 1% bovine serum albumin (BSA) in phosphate buffered saline (PBS) for 2 hours at RT. After blocking, plasma samples (1:1000 dilution in 1% BSA in PBS with 2mM EDTA) were added and incubated overnight at 4°C. Anti-DNA-HRP from Cell Death Detection ELISA kit (clone MCA-33; Roche) was added as secondary antibody for 1.5 hour at RT. The reaction was developed with 3,3′,5,5′ tetramethylbenzidine (TMB; BD Biosciences) and ended by the addition of 2N sulfuric acid. Known concentrations of MPO-DNA complexes (rhMPO, R&D Systems; Calf thymus DNA, Trevigon) were used to construct a standard curve. Plasma levels of human formyl methionine (fMet) were analyzed using a commercial ELISA kit according to manufacturer’s instructions (My BioSource Inc., San Diego, CA, USA). Absorbance for all ELISA assays were measured at 450 nm with a Synergy plate reader (BioTek).

### Neutrophil Activation Assay

Neutrophils were isolated from healthy subjects by layering heparinized blood on Polymorphprep (Axis-Shield, Dundee, UK) density gradient, according to the manufacturer’s instructions, or as described previously (7). Red blood cells were lysed with RBC lysis buffer (BioLegend, San Diego, CA USA). For *in vitro* assays, neutrophils were re-suspended in serum-free RPMI-1640 medium (ThermoFisher). Neutrophils were plated at 2.5 - 3 × 10^5^ cells/well and were incubated with or without a selective inhibitor of FPR1, cyclosporin H (CsH, 5 μM) for 30 min prior to the addition of stimuli. Antibodies directed against the human FcγRII (CD32) (5 μg/ml; Caprico Biotechnologies, Norcross, GA USA) was also added for 30 min before addition of stimuli. As stimuli, R848 (2.5 μg/ml), SSc plasma having either high or low levels of fMet (n=15 each) and healthy control plasma (n=6) (1:50 dilution) from Cohort I were used and incubated with the neutrophils for an additional 2 hours. Non-activated neutrophils were used as negative controls. Approximately 90-95% of neutrophils were viable after neutrophil stimulation with plasma samples. Additionally, quantification of neutrophil DNA release (NETosis) were measured as described previously (26). No/low level of NET formation was evident from neutrophils incubated with HC and SSc plasma during the neutrophil activation assays. Neutrophil activation was assessed by analyzing cell surface expression of CD66b (clone G10F5, BioLegend) and CD11b (clone CBRM1/5, BioLegend) by flow cytometry. Data were analyzed by FlowJo (Tree Star Inc, Ashland, OR) and results were presented as relative mean fluorescent intensity (MFI) % of CD66b and CD11b relative to healthy controls (set as 100%). % Inhibition was calculated as 1-(plasma induced activation marker MFI-100)/(plasma induced activation marker MFI in presence of CsH-100) x 100. This value was further subtracted by 100 as a baseline based on healthy controls (set as 100%).

### Statistical Analysis

For sample sets with a non-Gaussian distribution, non-parametric tests, Mann‐Whitney U test and Spearman’s correlation test were used when applicable. For plasma-mediated neutrophil activation studies, Mann-Whitney U test or Wilcoxon’s paired test were performed. GraphPad Prism and SPSS software were used for the analysis. P values less than 0.05 were considered significant.

## Results

### Levels of Soluble Neutrophil Activation Markers Are Elevated in SSc Patients

**Figure 1:**
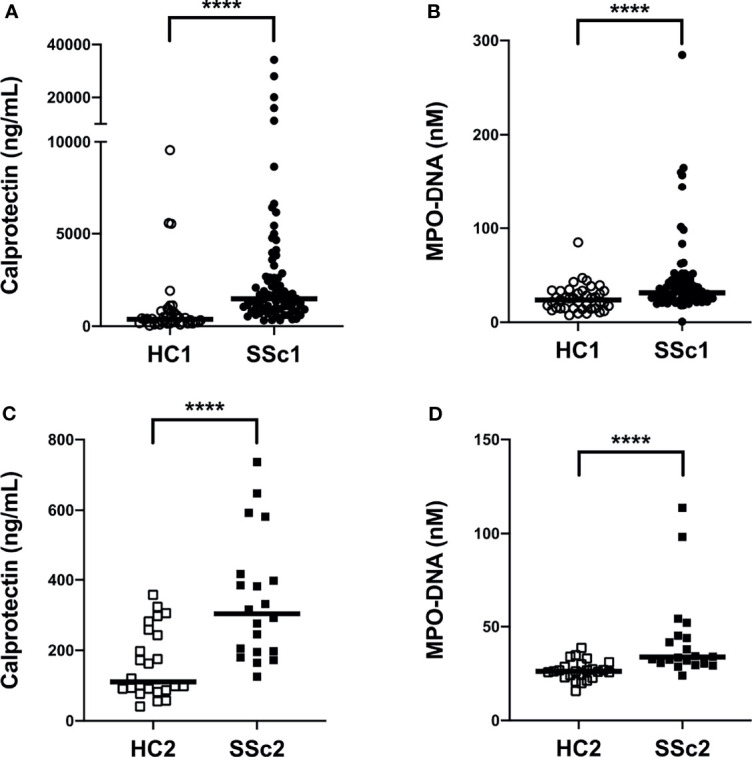
Increased plasma levels of neutrophil activation markers in systemic sclerosis (SSc) patients. **(A, C)** Levels of calprotectin (S100A8/A9) and **(B, D)** neutrophil extracellular traps (NETs, measured as myeloperoxidase-DNA complexes) were analyzed by ELISA in plasma samples of two cohorts of SSc patients and two cohorts of healthy controls (HC). Each symbol represents a single subject. **(A, B)** Cohort I: HC1, White circle (○); SSc1, Black circle (•). **(C, D)** Cohort II: HC2, White square (□); SSc2, Black square (▪). Bars represent the median and statistics were determined by Mann-Whitney U test with ****p<0.0001.

To investigate if neutrophil activation occurs in SSc, we analyzed levels of calprotectin (S100A8/A9) as well as NETs (MPO-DNA complexes) in plasma samples from a large cohort of patients with SSc (n=80, Cohort I, **Table 1**) as well as in a smaller validation cohort (n=20, Cohort II, **Table 1**) and compared them with levels found in healthy controls (n=40, Cohort I and n= 24, Cohort II). Levels of calprotectin (p<0.0001) and MPO-DNA complexes (p<0.0001) were elevated in both SSc cohorts, as compared to healthy controls (**Figure 1**). Additionally, there was correlation of calprotectin and MPO-DNA levels in both SSc cohorts (Cohort I: r=0.30, p=0.006; Cohort II: r=0.48, p=0.03, data not shown). These data clearly indicate neutrophils undergo marked activation and cell death in SSc.

### Association of Neutrophil Activation Markers With Clinical Variables in SSc Patients

**Figure 2:**
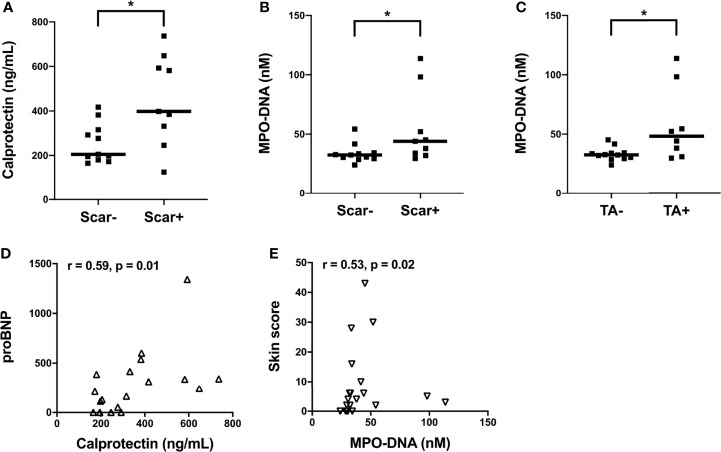
Associations of plasma levels of neutrophil activation markers with clinical parameters in SSc patients from Cohort II. **(A)** Plasma calprotectin levels in the presence and absence of scar tissue manifestation in SSc patients. Plasma NET (myeloperoxidase-DNA complexes) levels in the presence and absence of **(B)** scar tissue manifestation and **(C)** telangiectasia (TA) condition in SSc patients. **(A-C)** Cohort II: SSc2, Black square (▪). Shown are the correlation analysis between **(D)** calprotectin levels and brain natriuretic peptide (proBNP) (Δ) and **(E)** NET (myeloperoxidase-DNA complexes) levels and Skin score (∇) in SSc patients. Each symbol represents a single subject. Bars represent the median and statistics were determined by **(A-C)** Mann-Whitney U test with *p<0.05 and **(D, E)** Spearman’s correlation test.

Next, we assessed the clinical correlations of plasma calprotectin and NET levels in the clinically well-characterized SSc Cohort II. Due to limited clinical data on disease activity measures from Cohort I we could not assess their association to neutrophil biomarkers. Elevated levels of calprotectin and NETs in Cohort II SSc patients were correlated with vascular manifestations such as pitting scars (p<0.05, **Figures 2A, B**). In addition, SSc patients with telangiectasia had significantly higher levels of circulating NETs (p<0.05, **Figure 2C**) but not calprotectin (p=0.18, data not shown) as compared to patients without this manifestation. The plasma levels of calprotectin but not NETs positively correlated with their corresponding brain natriuretic peptide (proBNP) levels in the SSc patients in Cohort II (r= 0.59, p=0.01, **Figure 2D**). A positive correlation between circulating NET levels and skin score was also prominent in the Cohort II SSc patients (r=0.53, p=0.02, **Figure 2E**). Unexpectedly, no significant correlation was found between plasma calprotectin levels and skin score in the SSc patients (r=0.34, p=0.14, data not shown). With regards to disease duration, none of the neutrophil biomarkers correlated with disease duration in either of the cohorts. Additionally, no significant differences in neutrophil biomarker levels were present in patients with dcSSc as compared to lcSSc in either of the cohorts. Moreover, presence of common autoantibodies in both Cohort I and Cohort II were assessed, and none of the autoantibodies were associated with presence of neutrophil activation markers at time-point of blood draw (data not shown). Thus, neutrophil activation was associated with several skin- and vascular-related disease phenotypes in SSc patients.

### Levels of N-Formyl Methionine Peptides Are Elevated in Patients With SSc

**Figure 3:**
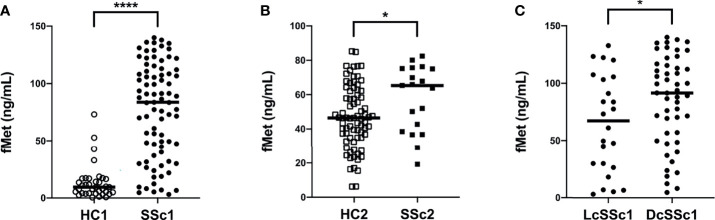
Increased plasma levels of mitochondrial protein fMet in SSc patients. **(A, B)** fMet levels were analyzed by ELISA in two cohorts of SSc patients and healthy controls (HC). **(C)** SSc patients from Cohort I were stratified based on disease phenotype, lcSSc and dcSSc, and assessed for fMet levels. Each symbol represents a single subject. **(A)** Cohort I: HC1, White circle (○); SSc1, Black circle (•). **(B)** Cohort II: HC2, White square (□); SSc2, Black square (▪). Bars represent the median and statistics were determined by Mann-Whitney U test with *p<0.05, ****p<0.0001.

We have recently demonstrated that patients with RA have elevated plasma levels of mitochondrial-derived N-formyl methionine peptides (fMet), a potent neutrophil agonist promoting neutrophil chemotaxis and activation, including NET formation (28). However, whether patients with SSc have elevated levels of fMet, and their potential contribution to neutrophil-mediated inflammation is not known. To address this, we analyzed levels of fMet in plasma samples from both SSc cohorts. As compared to healthy controls, patients with SSc had significantly higher levels of fMet in plasma (p<0.0001 and p=0.03, respectively, **Figures 3A, B**). Further, increased fMet levels were present in patients with dcSSc as compared to lcSSc in Cohort I (p=0.04, **Figure 3C**). With regards to associations with clinical variables, unlike neutrophil activation markers, fMet levels did not associate with clinical characteristics (pitting scar, skin score, proBNP, TA) in Cohort II SSc patients. Additionally, levels of fMet did not correlate with disease duration (r=-0.08, p=0.49; and r=-0.32, p=0.19 respectively, data not shown) and common autoantibodies in either of the cohorts.

### Association of Neutrophil Activation Markers With Levels of N-Formyl Methionine Peptides

**Figure 4:**
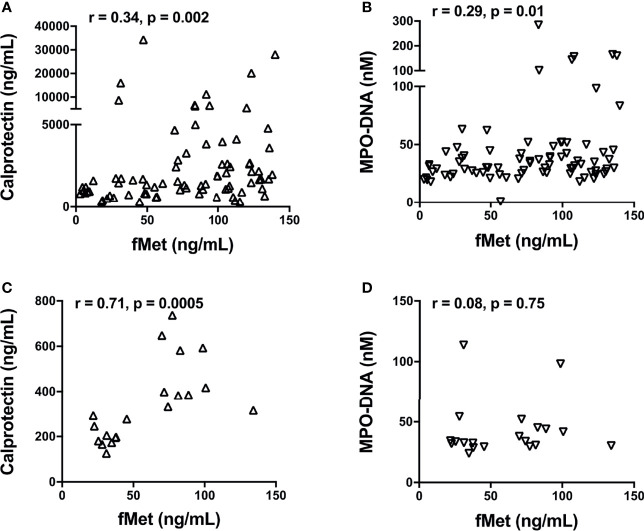
Levels of fMet in SSc patients associate with neutrophil activation markers. Levels of fMet, calprotectin, and NETs (myeloperoxidase-DNA complexes) were analyzed by ELISA. Shown are the correlation analyses between **(A)** calprotectin (Δ) and **(B)** NETs (∇) with fMet levels in SSc patients from Cohort I. **(C)** Calprotectin (Δ) and **(D)** NETs (∇) correlation with fMet levels is shown in SSc patients from Cohort II. Each symbol represents a single subject. Statistics were determined by Spearman’s correlation test.

As fMet is known to induce neutrophil activation through FPR1, we next asked whether plasma levels of fMet were associated with neutrophil activation markers in patients with SSc. In SSc Cohort I, levels of calprotectin (r=0.34, p=0.02, **Figure 4A**) and NETs (r=0.29, p=0.01, **Figure 4B**) correlated significantly with levels of fMet, suggesting fMet-mediated neutrophil activation in these patients. Similar findings were seen in Cohort II, with levels of calprotectin correlating with levels of fMet (r=0.71, p=0.005, **Figure 4C**). However, in contrast, no significant correlation was found between levels of NETs and fMet in Cohort II (r=0.08, p=0.75, **Figure 4D**). In all, neutrophil activation markers are associated with presence of the neutrophil agonist, fMet, in patients with SSc.

### N-Formyl Methionine Peptides Promote Neutrophil Activation Through FPR1 in SSc

**Figure 5:**
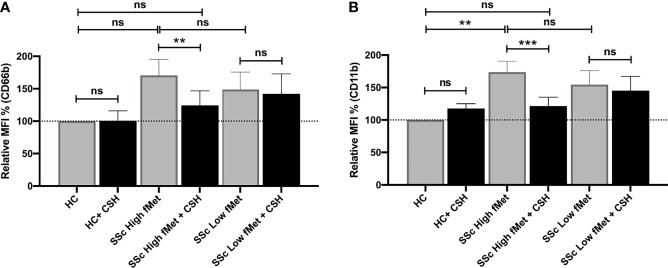
Plasma fMet can induce neutrophil activation in an FPR1-dependent manner. **(A, B)** Plasma from healthy controls, SSc patients containing high fMet levels and low fMet levels were incubated for 2 hours with healthy neutrophils in the presence or absence of Cyclosporine H (CsH) and assessed for the capacity to induce upregulation of neutrophil activation markers **(A)** CD66b and **(B)** CD11b. Bar graphs (means ± SEM) indicate the relative MFI % of CD66b and CD11b which was calculated as CD66b and CD11b MFI induced by stimuli divided by healthy control x 100. All analyses were performed using plasma from patients in Cohort I (HC, n=6; plasma with high fMet, n=15; plasma with low fMet, n=15). Data are combined from two independent experiments. Statistics were performed by Mann-Whitney U test or Wilcoxon’s paired test with **p<0.01, ***p<0.001 and ns, non-significant.

Considering the association between elevated levels of fMet and neutrophil activation markers in patients with SSc, we asked whether circulating levels of fMet could promote *de novo* neutrophil activation in an experimental model system. Purified fMet (fMLP: N-Formyl-Met-Leu-Phe) has been previously shown to activate neutrophils in a dose-dependent manner (35). We have also performed dose response curves using fMLP and saw a dose dependent neutrophil activation (CD66b activation marker, **Supplemental Figure 1**). Additionally, we also quantified NET formation induced by purified fMet (fMLP) at various concentrations and did not observe fMLP-mediated NET formation similar to previous studies where fMLP did not induce NET formation (36) (**Supplemental Figure 2**). Thus, purified fMet like molecules such as fMLP does not contribute to NETosis but induces neutrophil activation which were further assessed in this study. Neutrophils, isolated from healthy individuals, were incubated with plasma samples from Cohort I SSc patients and assessed for capacity to induce neutrophil activation by analyzing cell surface expression of CD66b and CD11b by flow cytometry (**Figures 5A, B**). Plasma samples from SSc patients having either high or low levels of fMet were used for this study. SSc plasma with high fMet levels induced marked neutrophil activation, illustrated by upregulation of CD66b (p=0.02) and CD11b (p<0.001) as compared to healthy controls. Intriguingly, increased neutrophil activation was also observed with SSc plasma with low fMet levels similar to SSc plasma with high fMet levels (CD66b; p=0.4 and CD11b; p=0.5).

To investigate whether the capacity of plasma to induce neutrophil activation was dependent on fMet, neutrophils were pre-incubated with the specific fMet receptor FPR1 antagonist Cyclosporine H (CsH) prior to addition of plasma samples. We and others have previously shown the specificity of CsH as an FPR1 inhibitor (28, 37). For patients with high levels of fMet, plasma-mediated neutrophil activation was reduced in the presence of the FPR1 inhibitor (CD66b; p<0.01 and CD11b; p<0.0001). Percentage of inhibition of CD66b and CD11b markers in presence of CsH were 66.7% and 71.6% respectively, suggesting neutrophil activation being partly mediated through the fMet/FPR1 pathway in these patients. Additionally, levels of plasma-mediated neutrophil activation in presence of CsH from patients with high levels of fMet were similar to those observed in healthy controls (CD66b; p=0.7 and CD11b; p=0.5) emphasizing the contribution of fMet in plasma to neutrophil activation. In contrast to plasma samples with high fMet levels, the FPR1 antagonist CsH could not inhibit neutrophil activation induced by SSc plasma samples with low fMet levels (CD66b; p=0.5 and CD11b; p=0.5). These results suggest that neutrophil activation is primarily driven by the fMet/FPR1 pathway in patients with high levels of circulating fMet, whereas other mechanisms are operating in low fMet disease states to activate neutrophils.

**Figure 6:**
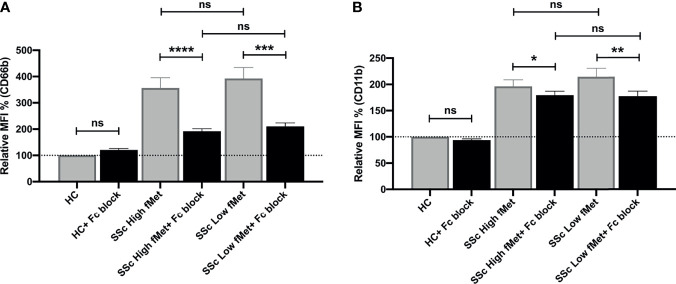
Immune complex mediated neutrophil activation by plasma from SSc patients. **(A, B)** Plasma from healthy controls, SSc patients containing high fMet levels and low fMet levels were incubated for 2 hours with healthy neutrophils in the presence or absence of FcyR blocking antibody (Clone IV.3) and assessed for the capacity to induce upregulation of neutrophil activation markers **(A)** CD66b and **(B)** CD11b. Bar graphs (means ± SEM) indicate the relative MFI % of CD66b and CD11b which was calculated as CD66b and CD11b MFI induced by stimuli divided by healthy control x 100. All analyses were performed using plasma from patients in Cohort I (HC, n=6; plasma with high fMet, n=15; plasma with low fMet, n=15). Data are representative from two independent experiments. Statistics were performed by Mann-Whitney U test or Wilcoxon’s paired test with *p<0.05; **p<0.01, ***p<0.001, ****p<0.0001 and ns, non-significant.

As fMet levels did not fully explain the neutrophil-activating capacity of plasma, we further assessed if immune complexes (ICs) contributed to the possible activation of neutrophils in SSc patients. We performed neutrophil activation experiments using a FcyRIIA blocking antibody (Clone IV.3) and found a significant decrease in neutrophil activation induced by SSc plasma containing high fMet levels (CD66b; p<0.0001 and CD11b; p=0.02) as well as low fMet levels (CD66b; p=0.0002 and CD11b; p=0.006) (**Figures 6A, B**). The percentage of inhibition of CD66b markers from high and low fMet levels SSc plasma by FcyR blocking antibody were 65.2% and 63.3% respectively, and for CD11b markers from high and low fMet levels SSc plasma by FcyR blocking antibody were 18.4% and 33.5% respectively. These observations suggest blocking FcyR significantly abrogates circulating IC-mediated neutrophil activation from the SSc plasma. Thus, both fMet and ICs may contribute to neutrophil activation in SSc.

## Discussion

Owning to its unknown etiology, SSc remains a poorly understood autoimmune disease and this presents one of the greatest challenges to both investigators and physicians. Despite thorough investigations, other than SSc-specific autoantibodies, few biomarkers are validated and/or widely used diagnostically or clinically (38). With respect to early immune cells in SSc pathogenesis, neutrophils are instrumental, infiltrating the lesions and participating in chronic inflammation and fibrosis (5, 39). Although neutrophils are implicated in SSc pathogenesis, neutrophil biomarkers have not been carefully investigated. In the current study we performed a comprehensive analysis on neutrophil activation biomarkers and assessed the role of extracellular mitochondrial components as potential neutrophil activating agents in SSc. Our findings indicate elevated levels of neutrophil activation markers and cell death in SSc. Neutrophil biomarkers associated with SSc disease phenotypes offering potential clinical value to these biomarkers by improving the capacity to monitor disease.

Neutrophils have several effector functions crucial for eliminating invading pathogens. In contrast, uncontrolled activation of neutrophils, including release of inflammatory mediators such as calprotectin can lead to chronic inflammation and immune dysregulation. Calprotectin is primarily produced by neutrophils comprising approximately 45% of its cytoplasmic content (40) and can be released into the extracellular environment upon degranulation. Once extracellular, calprotectin is an efficient damage-associated molecular pattern (DAMP) inducing inflammation by signaling through Toll-like receptor 4 and receptor for advanced glycation end products (RAGE) (41, 42). Thus, in view of its vital role in the physiology of inflammation, calprotectin is a valuable candidate as a neutrophil activation biomarker for inflammation-associated diseases.

Calprotectin (S100A8 and S100A9), either as homodimers, or heterodimer (e.g. calprotectin), have been found at elevated levels in plasma, sera (20, 43), feces (19), saliva (44), BAL fluids (21), and skin (43) of SSc patients. These studies reflect that SSc is not only a skin disease but also affects several visceral organs. Thus, it’s possible that neutrophil infiltrates might be present in skin and/or other organs affected by the disease, which needs to be further investigated. With regards to neutrophil activation markers, elevated calprotectin levels in the BAL fluid (21) and serum (20, 43) were associated with extensive lung fibrosis and anti-topoisomerase I (ATA) positivity in the SSc patients. Moreover, fecal calprotectin levels corelated with clinically important features of gastrointestinal (GI) disease and has been explored as a possible biomarker of GI disease in SSc (19). In the same study, plasma calprotectin levels were measured which corelated with systemic inflammation markers like C-reactive protein (CRP) (19). In the current study, we were able to validate elevated levels of calprotectin in SSc patients as well as add to previous findings regarding its potential clinical applications. In particular, our study showed increased calprotectin levels in patients with vasculature-related manifestations like cutaneous pitting scars. Additionally, correlation of calprotectin with proBNP was observed which has been shown to be a useful biomarker for assessing extent of skin fibrosis, degree of restricted pulmonary involvement and a diagnostic marker for pulmonary arterial hypertension (PAH) in SSc (38, 45). These observations further highlight the pathological significance of calprotectin levels in SSc and a promising non-invasive biomarker of SSc.

Effector functions of neutrophils like ROS production has been linked to pathogenesis of SSc. Generation of ROS is crucial for NET formation, a neutrophil cell death process and a well-known biomarker in diseases like RA and SLE (46). Similar to calprotectin, we found elevated levels of NETs (MPO-DNA complexes) in plasma samples of SSc patients, consistent with prior work (22). This study also observed elevated NET levels in the plasma of active SSc patients (22). Moreover, our study showed correlation of NETs with skin score, a measure of SSc disease progression over time, offering a potential clinical value to NET levels. Association of NETs with vascular manifestations (pitting scars, telangiectasia, nail fold capillary abnormalities) were also observed in our study and the above-mentioned study indicating NETs as a reliable marker to detect the presence of vascular involvement in active SSc patients (22). Elevated level of circulating NETs observed in SSc could be due to increased formation of NETs. Consistent with the hypothesis, prior findings have demonstrated increased capacity of SSc neutrophils to undergo NET formation after stimulation with autologous serum. In addition, neutrophils from SSc patients with severe vascular complications were significantly more prone to releasing NETs compared to other SSc patients (47). Other mechanisms contributing to elevated levels of circulating NETs such as decreased degradation/clearance of NETs in SSc patients are not reported yet and need further investigation. Thus, NET formation might represent a new pathophysiological as well as potential SSc biomarker.

The initial trigger(s) of neutrophil activation in SSc patients remain to be determined. In a recent study, microparticles released from activated platelets expressing the DAMP HMGB1 were abundantly found in blood of SSc patients (22). These microparticles resulted in neutrophil activation and generation of NETs and were further ablated in presence of BoxA, a competitive inhibitor of HMGB1, indicating a platelet-microparticle specific neutrophil activation *via* HMGB1 (22). Although not in the context of SSc, our group has recently demonstrated extracellular mitochondrial N-formyl methionine (fMet) peptides abundantly present in RA patients (28) as well as in systemic vasculitides (Michailidou et al., under revision) suggesting fMet-mediated neutrophil activation possibly being a central process in several autoimmune inflammatory conditions. Whether SSc patients have elevated levels of extracellular mitochondrial fMet and their role in neutrophil-mediated inflammation has so far not been investigated. Consistent with the RA study, elevated level of fMet peptides were found in SSc patients prompting us to investigate its role in neutrophil activation. Additionally, increased levels of fMet were observed in DcSSc as compared to LcSSc patients, suggesting fMet levels might play an important role in the pathogenesis of DcSSc and could be useful serological marker for evaluating type of SSc disease. Mitochondrial fMet peptides can be sensed by N-formyl peptide receptor FPR1, which has high affinity for fMet, and is expressed on various host cell types but most strongly on neutrophils. Activation of neutrophils by fMet *via* FPR1 triggers a wide variety of downstream effector functions including chemotaxis, degranulation, ROS production, and phagocytosis bridging an association between mitochondrial fMet proteins, FPR1 and neutrophils (48).

Association between mitochondrial proteins and neutrophil activation was shown in our recent study of RA patients (28). Similarly, we found levels of fMet associating with neutrophil activation markers like calprotectin and NETs in SSc patients, supporting the hypothesis of fMet-mediated neutrophil activation. Given that correlations do not inform on causality, we performed neutrophil stimulation studies to identify circulating factors such as fMet present in plasma that are able to activate neutrophils *in vitro.* Our data demonstrate that plasma from SSc patients having high fMet levels in circulation had increased ability to induce activation of neutrophils from healthy blood donors. Blockade of FPR1 by cyclosporin H (CsH) suggested that circulating fMet activates FPR1 signaling in SSc patients and contribute significantly to the immune activation of neutrophils. These observations warrant exploring the inhibition of FPR1 signaling as a potential novel pharmaceutical intervention in SSc, a disease which is in dire need of novel therapeutics. Importance of FPR1 signaling has been previously shown in tissue fibrosis, the hallmark of SSc. FPR1 signaling has been shown to be crucial for neutrophil recruitment to the lung and support fibrosis in an *in vivo* mouse model of SSc (49). FPR1 is also expressed on human fibroblasts and signaling *via* FPR1 in SSc patients has been shown to promote transition of fibroblast-to-myofibroblasts and extracellular matrix deposition leading to tissue fibrosis (50). Thus, fMet proteins critical to FPR1 signaling and neutrophil activation might be an important mechanism contributing to SSc pathogenesis and a potential therapeutic target. There are several potential sources of extracellular mitochondria, including platelet activation, neutrophil death, and tissue damage (26, 27). Which, if any, of these mechanisms operate in SSc to promote release of extracellular mitochondria is under current investigation.

Adding to the complexity of SSc, in contrast to our hypothesis we found activation of neutrophils even upon stimulation with a subgroup of SSc patients having low fMet levels in plasma. The activation of neutrophils did not decrease upon blockage of FPR1 indicating the neutrophil activation was not driven by fMet/FPR1 interactions but due to other circulating factors present in the plasma samples. Among the circulating factors, immune complexes (ICs) containing SSc-specific autoantibodies engaging with nucleic acid or DNA/RNA binding proteins are shown to elicit proinflammatory and profibrotic effects on fibroblasts (51). The pathogenicity of the SSc-ICs has been suggested to be mediated by interaction with Toll-like receptors (TLRs) *via* nucleic acid fragments. Additionally, circulating ICs contributing to neutrophil activation has been shown in autoimmune diseases like SLE (52). However, in the current study, presence of SSc autoantibodies towards intracellular antigens did not associate with levels of circulating NETs (data not shown), but neutrophil activation upon stimulation with SSc plasma was abrogated upon blocking of Fcy receptor, the binding sites of ICs. Thus, fMet is an important driver of neutrophil activation and inflammation in a subgroup of SSc patients with high levels of fMet in circulation. However, other mechanisms, potentially driven through ICs, are operating in patients with low levels of fMet. These results further highlight the heterogeneity of the disease and the need for patient stratification or personalized medicine approach, as different patients might have different immunological pathways activated and might not necessarily benefit from the same treatment.

The limitation of our study includes limited clinical data for SSc Cohort I, as well as lack of longitudinal data, which would have allowed us to assess the prognostic utility of the neutrophil biomarkers. Another limitation of our study includes conducting experiments on neutrophil activation alone *via* fMet/FPR1 signaling. Although FPR1 is expressed strongly on neutrophils, other innate immune cells like monocytes and macrophages also express FPR1 warranting analyzing these cell types which are also implicated in early SSc pathogenesis (5).

In conclusion, our data for the first time demonstrated that levels of fMet are elevated in the circulation of SSc patients and implicate an important role for the mitochondrial component fMet in promoting neutrophil-mediated activation through FPR1 in SSc. Our data also support the clinical value of neutrophil biomarkers and fMet in monitoring SSc disease, although these observations need to be validated in larger patient cohorts as well as in appropriate animal models. We propose fMet-mediated signaling as a potential therapeutic target promoting anti-inflammatory effects in SSc by ameliorating neutrophil based inflammation.

## Data Availability Statement

The original contributions presented in the study are included in the article/**Supplementary Material**. Further inquiries can be directed to the corresponding author.

## Ethics Statement

The studies involving human participants were reviewed and approved by Institutional Review Board of Fred Hutchinson Cancer Research Center (part of the University of Washington Consortium) and Lund University #2010/544. The patients/participants provided their written informed consent to participate in this study.

## Author Contributions

RK and CL conceived the study. RK, BD, and CL designed experiments, analyzed data, and interpreted results. RK, RS, BD, TW, and MF performed experiments. JN, RH, and MF provided materials and clinical cohorts. RK wrote the manuscript and RH, JN, MF, RS, BD, and CL critically reviewed the manuscript. All authors reviewed and approved the manuscript.

## Funding

This work was supported by grants from the Arthritis National Research Foundation (#632002) to CL and NIH grant R01 AI-41721 and a Scleroderma Foundation grant to JN.

## Conflict of Interest

The authors declare that the research was conducted in the absence of any commercial or financial relationships that could be construed as a potential conflict of interest.

## Publisher’s Note

All claims expressed in this article are solely those of the authors and do not necessarily represent those of their affiliated organizations, or those of the publisher, the editors and the reviewers. Any product that may be evaluated in this article, or claim that may be made by its manufacturer, is not guaranteed or endorsed by the publisher.
