# Supplementary material for: N-Formyl Methionine Peptide-Mediated Neutrophil Activation in Systemic Sclerosis
Source: Front Immunol. 2022 Jan 5;12:785275. doi: 10.3389/fimmu.2021.785275 (PMC8766990; doi:10.3389/fimmu.2021.785275)
Supplement: Supplementary file 1 [file DataSheet_1.docx]

**Supplemental Figure 1:**

**Supplemental Figure 1. Purified fMet (fMLP: N-Formyl-Met-Leu-Phe) induce neutrophil activation in a dose-dependent manner.** Healthy neutrophils were incubated in the presence or absence of fMLP at various concentrations for 2 hrs and assessed for the capacity to induce upregulation of neutrophil activation marker CD66b. Bar graphs (means ± SEM) indicate the MFI of CD66b (n=2). R848 (2.5 ug/ml) were used as positive controls.

**Supplemental Figure 2:**

**Supplemental Figure 2. Purified fMet (fMLP: N-Formyl-Met-Leu-Phe) do not induce NETosis from neutrophils.** Healthy neutrophils were incubated in the presence or absence of fMLP at various concentrations for 4 hrs and assessed for the capacity to induce NETosis. Bar graphs (means ± SEM) indicate the concentration of DNA released from neutrophils (n=2). PMA and Calcium Inophore A23187 were used as positive controls.
